# Identification and Characterization of Preferred DNA-Binding Sites for the *Thermus thermophilus* HB8 Transcriptional Regulator TTHA0973

**DOI:** 10.3390/ijms20133336

**Published:** 2019-07-07

**Authors:** James Shell Cox, Kristi Moncja, Mykala Mckinnes, Michael W. Van Dyke

**Affiliations:** Department of Chemistry and Biochemistry, Kennesaw State University, Kennesaw, GA 30144, USA

**Keywords:** bioinformatics, biolayer interferometry (BLI), electrophoretic mobility shift assay (EMSA), extremophile, type IIS restriction endonuclease

## Abstract

Advances in genomic sequencing have allowed the identification of a multitude of genes encoding putative transcriptional regulatory proteins. Lacking, often, is a fuller understanding of the biological roles played by these proteins, the genes they regulate or regulon. Conventionally this is achieved through a genetic approach involving putative transcription factor gene manipulation and observations of changes in an organism’s transcriptome. However, such an approach is not always feasible or can yield misleading findings. Here, we describe a biochemistry-centric approach, involving identification of preferred DNA-binding sequences for the *Thermus thermophilus* HB8 transcriptional repressor TTHA0973 using the selection method Restriction Endonuclease Protection, Selection and Amplification (REPSA), massively parallel sequencing, and bioinformatic analyses. We identified a consensus TTHA0973 recognition sequence of 5′–AACnAACGTTnGTT–3′ that exhibited nanomolar binding affinity. This sequence was mapped to several sites within the *T. thermophilus* HB8 genome, a subset of which corresponded to promoter regions regulating genes involved in phenylacetic acid degradation. These studies further demonstrate the utility of a biochemistry-centric approach for the facile identification of potential biological functions for orphan transcription factors in a variety of organisms.

## 1. Introduction

Transcriptional regulation is the primary means by which most organisms, both prokaryotic and eukaryotic, control gene expression. Transcriptional regulation occurs through the combinatorial recognition of specific DNA elements by sets of proteins, commonly referred to as transcription factors, that affect access of RNA polymerase to gene promoter regions and rates of productive transcription. Understanding the sets of genes regulated by a particular transcription factor, or regulon, provides insights into the biological function of this protein and its role in an organism’s physiology.

With the advent of massively parallel sequencing techniques, the genomes of hundreds of thousands of organisms are presently known [1]. Within each, simple bioinformatic approaches can be used to identify dozens to hundreds of potential transcription factors, primarily based on their sequence homology with known transcription factors. However, beyond extensions based on work in evolutionarily related organisms, it is not yet possible to predict the DNA sequences recognized by a putative transcription factor or the genes that it regulates solely from genomic sequence information. Such knowledge still requires empirical data.

In organisms possessing powerful genetic tools, a forward approach involving transcription factor gene manipulation and molecular changes in gene expression can be employed. Comparing potential regulatory regions among the genes most affected may allow the identification of a consensus DNA element involved in their regulation. This can then be directly tested in vitro by a variety of biophysical means to validate transcription factor-DNA interactions. Scanning for consensus sequences throughout an organism’s genome, particularly those intergenic regions most likely involved in promoting transcription, can yield a panel of genes potentially regulated by a particular transcription factor. Bioinformatic analyses of these genes and downstream members of their operons, ranging from identification of homologous domains with known functions in their encoded proteins to phenotypic changes in response to environmental changes, can provide important clues as to the biological roles of an unknown transcription factor. Such an approach has been aptly demonstrated in the model organism *Escherichia coli* and can be seen in the wealth of information presently available on its transcription factors and the genes they regulate, as exemplified by the database RegulonDB [2]. Such provides a paradigm for understanding transcriptional regulatory networks in the host of organisms for which genomic information is now available.

Unfortunately, genetic tools may be limited for some organisms, making the aforementioned forward genetic approach for determining putative transcription factor function challenging. One case in point is the extremely thermophilic bacteria *Thermus thermophilus* HB8. Originally isolated from the Izu-Mine hot spring in Kawazu, Japan [3], it has been adopted as the model organism for the Structural-Biological Whole Cell Project, with the goal of understanding all biological phenomena in a cell through the structure of its biomolecules [4]. *T. thermophilus* HB8 is postulated to have 2226 genes in its genome, of which 1214 can be categorized by homology with regards to the potential biological functions of their encoded products. However, while tools such as microarray expression screening and state-of-the-art X-ray crystallography have been widely applied to its proteins, the polyploid nature of *T. thermophilus* has complicated genetic approaches towards understanding biological questions in this organism [5].

Our laboratory has developed an alternative, biochemistry-centric approach for determining the possible biological functions of putative transcription factors. First, we use the combinatorial selection method, Restriction Endonuclease Protection, Selection and Amplification (REPSA), massively parallel sequencing, and de novo motif discovery to determine a consensus DNA-binding sequence for a putative transcription factor [6,7,8]. This is then followed by motif scanning within the subject genome and bioinformatic analyses to identify potential regulated genes and their possible biological functions. Whenever possible, each step is validated by available means, ranging from biophysical characterization of transcription factor binding to target sequences to microarray gene expression profile data. To test the utility of our approach, we have chosen to investigate four putative *T. thermophilus* HB8 transcription factors, TTHA0101, TTHA0167, TTHA0973, and TTHB023. All are structurally related, possessing an N-terminal α-helix-turn-α-helix motif (pfam00440) characteristic of the archetype TetR transcriptional repressor protein in *E. coli*, which is responsible for their sequence-specific DNA binding. Each has been previously investigated through other means with regards to their DNA-binding specificity and regulatory profiles [9,10,11,12]. In the present paper, we present a REPSA-based investigation into the DNA-binding specificity and possible genomic targets of the TTHA0973 protein, comparing our results with those previously obtained by more conventional methods. This study provides us with a better understanding of the strengths and weaknesses of a biochemistry-centric approach for investigating transcription factors and will help shape future studies on other uncharacterized, orphan transcription regulatory proteins.

## 2. Results

### 2.1. REPSA Selection of TTHA0973-Binding DNAs

**Figure 1:**
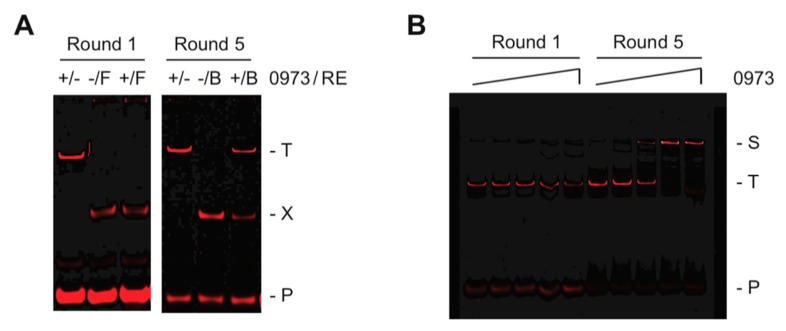
Selection and validation of TTHA0973-binding DNA sequences. (**A**) Shown are IR fluorescence images of restriction endonuclease cleavage protection assays made during Round 1 and Round 5 of REPSA selection with 17 nM TTHA0973 protein. The presence (+) or absence (−) of TTHA0973 and IISRE FokI (F) or BpmI (B) is indicated above each lane. The electrophoretic mobility of the intact (T) and cleaved (X) selection template, as well as the IRD7_ST2R primer (P), are indicated at the right of the figure. (**B**) Shown are IR fluorescence images of electrophoretic mobility shift assays made with DNA mixtures obtained following Round 1 (left lanes) and Round 5 (right lanes) of REPSA selection incubated with increasing concentrations of TTHA0973 protein (from left to right: 0, 0.8, 8, 80, or 800 nM TTHA0973). The electrophoretic mobility of a single protein-DNA complex (S) as well as uncomplexed ST2R24 selection template (T) and IRD7_ST2R primer (P) are indicated at the right of the figure.

To determine the preferred DNA-binding sites for the orphan transcription factor TTHA0973, we have chosen to use the selection method REPSA to screen a library of billions of possible sequences with purified recombinant TTHA0973 protein. ST2R24, our standard selection template, contains a central randomized 24-bp sequence flanked by defined DNA sequences, which serve the dual purpose of providing sites for DNA-binding by our IISRE probes and primer annealing during PCR amplification and has been previously described [7]. REPSA was initiated with 42 fmoles ST2R24, which provides a good representation of all possible 16-bp recognition sequences. Five rounds of REPSA were performed, three with the IISRE FokI followed by two with the IISRE BpmI. As shown in Figure 1A (left panel), the population of DNAs following one round of REPSA selection were not resistant to IISRE cleavage when TTHA0973 was present. However, after five rounds of selection (right panel), the resulting DNAs were resistant to IISRE cleavage in a TTHA0973-dependent manner, suggesting that preferred TTHA0973-binding sequences had been obtained. This was confirmed using an independent DNA-binding assay, EMSA, which demonstrated quantitative conversion of Round 5 DNAs to a slower mobility species when incubated with 80 nM TTHA0973 (Figure 1B). No comparable change in mobility was observed with Round 1 DNAs, even at the highest TTHA0973 concentration investigated (800 nM), suggesting that most all Round 5 DNAs contained specific TTHA0973 binding sites. Taken together, our data indicated that these DNAs would be worthy of massively parallel semiconductor sequencing to determine a consensus sequence for TTHA0973-DNA binding.

### 2.2. Identification and Characterization of a TTHA0973-Binding Consensus Sequence

**Figure 2:**
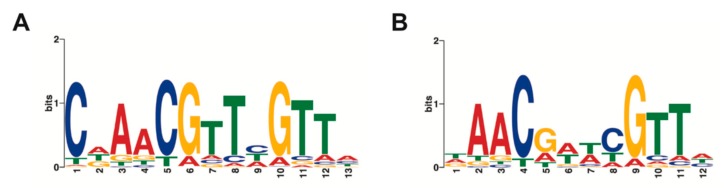
TTHA0973 consensus sequences. Sequence logos were determined using MEME software with inputs of 1000 Round 5 DNA sequences. (**A**) MEME performed with no filters. (**B**) Palindromic filter.

Round 5 REPSA-selected DNAs were subjected to fusion PCR to yield an amplicon library suitable for massively parallel semiconductor sequencing. Sequencing the TTHA0973 fusion library yielded 3,793,387 total bases, 2,756,517 ≥ Q20, and resulted in 77,936 reads of 49 bp mean length. This data in fastq format was further processed using our Sequencing1.java program and DuplicatesFinder v1.1, yielding 7453 unique sequences suitable for further analysis. Sets of 1000 sequences were input into web version 4.10.2 of Multiple Em for Motif Elicitation (MEME) using either no or a palindrome filter. Output in each case was position weight matrices, represented by sequence logos, corresponding to the top three sequence motifs identified. For TTHA0973, the best motif identified without a filter is the 13-mer shown in Figure 2A, while the best palindromic motif is the 12-mer shown in Figure 2B. Notably, all 1000 of the sequences investigated contained both the nonpalindromic and palindromic sequences, with statistical significance E-values of 1.3 × 10^−1951^ and 7.0 × 10^−1439^, respectively. These values are far more significant from those reported for most transcription factors (typically derived from two–10 sequences [2]) and can be considered with high confidence to be part of the consensus sequence for TTHA0973.

**Figure 3:**
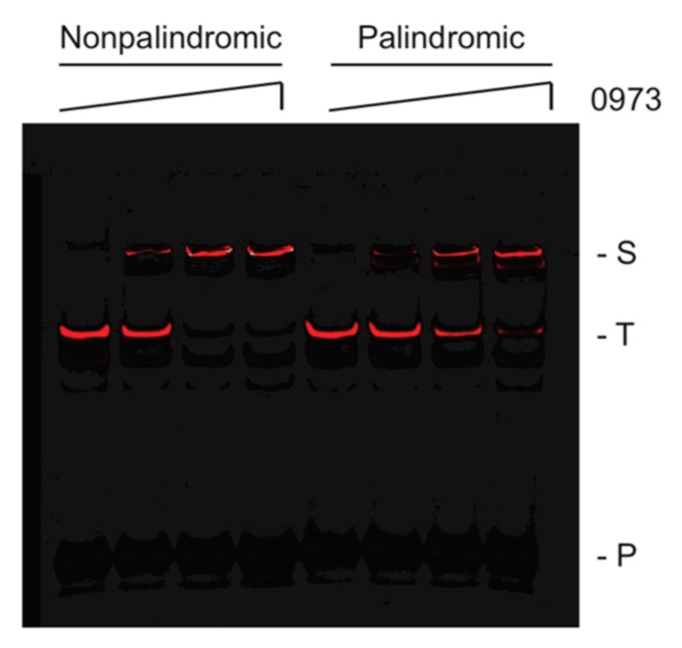
EMSA analysis of TTHA0973-binding to consensus sequences. Shown are IR fluorescence images of IRD700-labeled TTHA0973 nonpalindromic (left) or palindromic (right), as indicated, incubated with 0, 10, 30, or 100 nM TTHA0973 protein. (S) Protein-DNA complex, (T) uncomplexed DNA, (P) IRD7_ST2R primer.

To validate our TTHA0973 consensus sequences, IR fluorophore-labeled DNA probes were synthesized containing the most likely nucleotides at each position for both the 13-mer nonpalindromic sequence and the 12-mer palindromic sequence. These were incubated with increasing concentrations of TTHA0973 protein to affect binding and subjected to analysis by EMSA. As shown in Figure 3, both the nonpalindromic and palindromic probes exhibited slower mobility species consistent with specific binding. TTHA0973 binding to the longer, nonpalindromic sequence was slightly better, as seen from the increasing percentage of slower mobility species when the lowest concentration of TTHA0973 was present (10 nM). Conversely, the palindromic probe, while exhibiting a primary shifted species with equivalent electrophoretic mobility as the nonpalindromic probe, also demonstrated a minor species with slightly greater mobility at all TTHA0973 concentrations investigated. The cause for this minor species is not known but does not appear to be the preferred TTHA0973-palindromic DNA complex under our reaction conditions.

**Figure 4:**
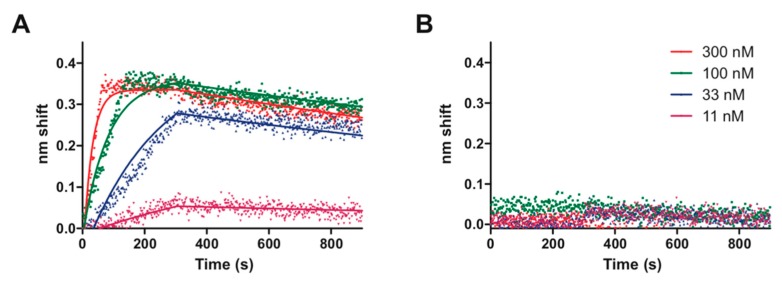
Biolayer interferometry analysis of TTHA0973 binding to DNA. Shown are raw traces (dots) and best-fit lines of TTHA0973 binding to (**A**) ST2_0973_R5_wt consensus DNA or (**B**) ST2_REPSAis control DNA. TTHA0973 concentrations investigated include 300 nM (red), 100 nM (green), 33 nM (blue), and 11 nM (magenta).

TTHA0973, having homology with TetR-family transcriptional regulators, might be expected to bind DNA as a homodimer and recognize a palindromic sequence. Such is supported by prior gel filtration column chromatography, which found that TTHA0973 preferentially exists as a homodimer in solution [11]. Our data so far are consistent with this supposition, as no additional proteins are required for TTHA0973 specific binding to both nonpalindromic and palindromic sequences. As all of our REPSA-selected DNAs contained both nonpalindromic and palindromic sequences, we devised a longer palindromic sequence, 5′–AACnAACGTTnGTT–3′, that possessed elements from both sets of sequences. This, then, was considered the consensus sequence for TTHA0973-DNA recognition and was the basis for all subsequent studies. To characterize this consensus, biotin-labeled DNA probes were synthesized containing this sequence or point mutants thereof (Table S1) and incubated with different concentrations of TTHA0973. These reactions were analyzed using biolayer interferometry, which provides real-time measurements of protein-DNA association and dissociation kinetics. Examples of these BLI experiments with probes containing the TTHA0973 consensus sequence (Figure 4A) or a control DNA (Figure 4B) are shown below. The consensus sequence demonstrated a concentration-dependent increase in interference change, with greatest effects occurring at 300 nM TTHA0973 (red dots) but still appreciable binding occurring at 11 nM (magenta dots). No observable interference change was observed with any TTHA0973 concentration investigated for the control DNA, indicating that nonspecific binding either does not occur under these conditions or cannot be detected.

Single-state association and dissociation kinetics were calculated from the data for TTHA0973 binding to its consensus sequence and are plotted as correspondingly colored lines in Figure 4A. Numerical values for association rate, dissociation rate, and apparent dissociation constant are also presented in Table 1. Notably, goodness-of-fit values for the binding of TTHA0973 to its consensus sequence were quite good (R^2^ = 0.9716), even though only four different TTHA0973 concentrations were investigated. Further characterization of the specificity of TTHA0973-DNA binding was explored through BLI experiments with selected point mutants of its consensus sequence. These data show that certain single mutations can greatly (5- to 10-fold) affect TTHA0973-DNA binding, whereas others had minimal effect. This information further confirms our sequence logos and will be of great importance when determining those native *T. thermophilus* HB8 sequences that should bind TTHA0973 with high affinity.

### 2.3. Identification of Potential TTHA0973-Binding Sites Within the T. thermophilus HB8 Genome

**Figure 5:**
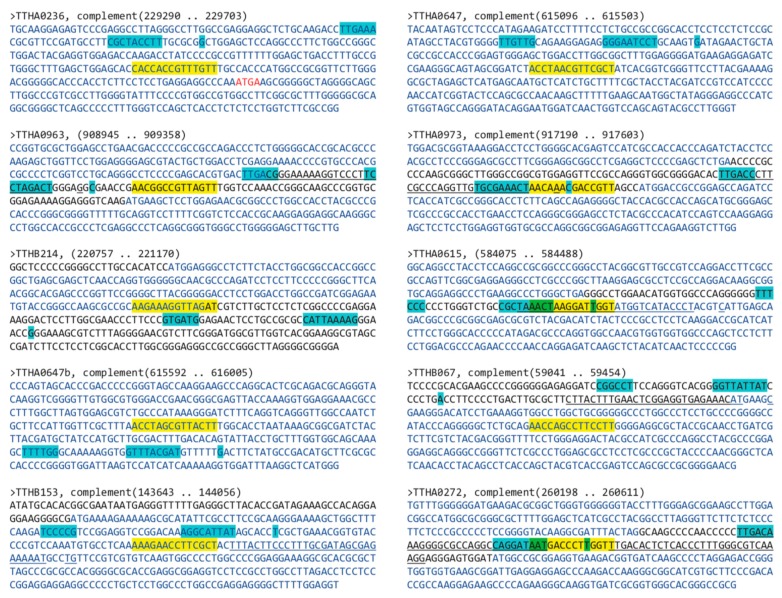
Bioinformatic identification of *T. thermophilus* HB8 promoters potentially regulated by TTHA0973. Shown are sequences +/− 200 bp of the TTHA0973-binding sequence identified through FIMO (see Table 2). Longest open reading frames with the same orientation as the target gene are indicated with blue nucleotides. Black nucleotides indicate intergenic regions; red nucleotides, overlapping open reading frames. Potential promoter elements (−30 and −10 boxes, +1 start site of transcription) identified using Softberry BPROM are indicated with cyan highlighting; those identified by U. Groningen PePPER by underlining. TTHA0973-binding sites are indicated with yellow highlighting. Overlapping TTHA0973-binding and core promoter elements are indicated by green highlighting.

Using the MEME Suite motif scanning program Find Individual Motif Occurrences (FIMO) and a 14-mer pseudopalindromic TTHA0973 consensus sequence to probe the *Thermus thermophilus* HB8 uid13202 210 database. Output was 142 motif occurrences with a P-value less than 0.0001. The top 10 unique occurrences, those whose *P*-values were ≤1.21 × 10^−5^, are shown in Table 2. To determine whether these were located in potential gene promoter regions, genomic *T. thermophilus* HB8 sequences ±200 bp of these sites were scanned using Softberry BPROM and University of Groningen Genome2D PePPER programs to identify potential bacterial core promoter elements. This analysis is shown in Figure 5. Several of the mapped TTHA0973 binding sites were found to be located in intergenic regions proximal to identifiable bacterial core promoter elements. These are best exemplified by the promoter regions upstream of genes *TTHA0963*, *TTHA0973*, *TTHA0615*, and *TTHA0272*. The other TTHA0973 sites were located within the open reading frames of postulated genes. However, these should not be formally excluded from further consideration, given that all contain possible cryptic core promoters that could be influenced by nearby TTHA0973 binding.

Similarly, while most bacterial transcriptional regulation often occurs at the level of operons, genes encoded by a common transcript but independently translated, one cannot exclude potential TTHA0973 regulation of transcripts originating from downstream genes. A search of TTHA0973 sites relative to postulated operons was performed using databases at the National Autonomous University of Mexico (ProOpDB) and BioCyc, results of which are listed in Table 2. Thus, only a small subset of our identified TTHA0973 binding sites in the *T. thermophilus* HB8 genome, *e.g*., *TTHA0963*, *TTHA0973*, *TTHA0615*, and *TTHA0272*, have most if not all of the characteristics expected for a TTHA0973 binding site capable of functionally regulating one or more downstream genes.

### 2.4. Validation of Potential TTHA0973-Regulated Genes in T. thermophilus HB8

While bioinformatic analyses may point to candidate genes for TTHA0973 regulation, it is important to determine whether this protein specifically and avidly binds its target sites within their promoters and regulates their transcription. To investigate the former, we used biolayer interferometry to determine the binding kinetics of TTHA0973 to each identified binding site. As shown in Table 3, TTHA0973 binding sites in or upstream of genes *TTHA0647*, *TTHA0963*, and *TTHA0973* demonstrated high affinity binding (K_D_ < 4 nM), *TTHA0236* and *TTHA0615* demonstrated intermediate affinity binding (8 to 38 nM), and *TTHA0647*b and *TTHB153* demonstrated relatively weak binding affinities (100 to 300 nM). Notably, the sites corresponding to *TTHB214*, *TTHB067*, and *TTHA0272* exhibited no apparent binding under our experimental conditions. Taken together, these data suggest that TTHA0973 would best be expected to occupy its high and potentially intermediate affinity binding sites under physiological conditions and that these and downstream genes would be most likely to be regulated by this protein.

To determine whether TTHA0973 may be involved in the regulation of these genes, we made use of publicly available expression profile data from TTHA0973-deficient strains of *T. thermophilus* HB8 [13]. Comparing expression profiles from three TTHA0973-deficient and three wild type *T. thermophilus* HB8 strains grown under identical conditions, we used NCBI GEO2R to rank tags according to their adjusted *P*-values. These are shown in Table 4. Note that many of the most significant candidates corresponded to genes having appreciable changes in expression (i.e., −2 > logFC > 2) between these two sets of strains, with both upregulation (positive logFC values) and downregulation (negative logFC values) being observed in this group. However, changes in expression levels do not always correspond to proof of direct transcriptional control, as levels in gene expression can be affected indirectly through the expression of additional transcription factors in response to depletion of a particular gene. Of the genes we had identified through our REPSA selection and bioinformatic analyses, only two, *TTHA0963* and *TTHA0973*, were in the top 100 GEO2R candidates. Both exhibited upregulation in TTHA0973-deficient strains, on the order of 4- to 100-fold, as compared to their wild type counterparts. Curiously, tags associated with *TTHA0973* were the most highly upregulated of all, even though this strain was supposedly TTHA0937 deficient. Such may reflect the number and location of the *TTHA0973* disruptions as well as the polyploid nature of *T. thermophilus* HB8 and a potential autoregulatory role for this transcriptional regulator [5,11].

### 2.5. Postulated Biological Role for TTHA0973 in T. thermophilus HB8

Taking together our data, including TTHA0973 binding site locations within the *T. thermophilus* HB8 genome, their relationship to gene promoters and defined operons, TTHA0973 binding affinity to these sites, and microarray expression profile data from wild type and TTHA0973-deficient *T. thermophilus* HB8 stains, it is possible to postulate those genes that are most likely regulated by TTHA0973. These are shown in Table 5. Prominent among them were genes in the two operons fronted by genes *TTHA0963* and *TTHA0973*. Applying bioinformatic data from available databases regarding the possible biological roles of their encoded proteins, we find that most of these genes could be involved in phenylalanine metabolism, particularly steps involved in the synthesis and degradation of phenylacetyl-CoA (*TTHA0966*, *TTHA0965*), in the conversion of phenylacetyl-CoA to 2-(1,2-epoxy-1,2-dihydrophenyl)acetyl-CoA (*TTHA0969*, *TTHA0970*, *TTHA0971*, *TTHA0972*), or degradation steps downstream of that (*TTHA0968*). A deficiency of TTHA0973 was found to upregulate genes in the *TTHA0963-67* operon 4- to 10-fold and in the *TTHA0973-68* operon 11- to 100-fold, indicating that TTHA0973 functions in both cases as a transcriptional repressor protein. Genes in the third operon potentially regulated by TTHA0973, *TTHA0615-6*, demonstrated low upregulation (< 2-fold) and low statistical significance when TTHA0973 was deficient. Thus, one does not have similarly high confidence that TTHA0973 also regulates expression of genes encoding an ATP-dependent Clp protease or the specific substrates for this protease.

## 3. Discussion

As a prelude to using our biochemistry-centric approach to characterize putative transcription factors in the extreme thermophile, *Thermus thermophilus* HB8, we chose to test this approach with four relatively well-characterized transcription factors. In the present report, we investigated the *T. thermophilus* HB8 transcription factor TTHA0973, which had been previously investigated by more conventional approaches [11]. Following REPSA selection, we isolated a library of DNA sequences that exhibited substantial TTHA0973-dependent cleavage inhibition by the type IIS restriction endonuclease BpmI. Sequencing these and performing MEME motif elicitation yielded consensus sequences that were merged to a single consensus sequence, 5′–AACnAACGTTnGTT–3′. In comparison, Sakamoto et al. defined the TTHA0973-regulated operons based on their homology to phenylacetic acid (PAA) clusters present in other organisms. Comparing their two promoter regions yielded a common pseudopalindromic sequence, 5′–CNAACGNNCGTTNG–3′. Notably, both the REPSA-selected and homology-derived consensus sequences are 14 bp and have elements in common, particularly the sequences 5′–CNAACG–3′ and 5′–CGTTNG–3′. However, with only two promoters identified in the latter approach, it is difficult to define the importance of all nucleotides in the consensus with regards to their role in TTHA0973-DNA recognition. For example, all REPSA-selected sequences had MEME-defined consensus sequences present, providing extremely high statistical significance *E*-values from 1.3 × 10^−1951^ to 7.0 × 10^−1439^. By comparison, a similar MEME analysis of the two promoter sequences did not yield any significant consensus sequences, given the limited number of sequences involved. Thus, with regards to understanding the DNA-binding specificity of a putative transcription factor, a selection-based approach like REPSA yielded a greater number of sequences with which to derive a more detailed consensus sequence. Curiously, Sakamoto et al. did perform a related selection method, genomic SELEX [16], to identify DNA fragments from the *T. thermophilus* HB8 genome that avidly bound TTHA0973 protein. What they obtained was 63 total clones, 24 unique. Of the latter, 17 contained only sequences within open-reading frames, and seven contained some intergenic regions. Focusing on the intergenic group, three unique clones were obtained that contained *TTHA0963* or *TTHA0973* promoter sequences, which helped form the basis of their consensus sequence for TTHA0973. The other clones had varying regions of homology to their defined consensus sequence, ranging from three to 10 base pairs. While REPSA and genomic SELEX are related combinatorial selection methods, they have different strengths and weaknesses [17]. In this particular circumstance, genomic SELEX did not provide a substantially greater understanding of TTHA0973-DNA binding specificity than what was obtained through homology studies alone and was far inferior to the extent of data obtained by REPSA.

Having identified TTHA0973-DNA consensus sequences by REPSA selection, we sought to validate these sequences through direct protein-DNA binding assays. EMSA, while qualitatively useful for determining different protein-DNA complexes, is not highly amenable for measuring kinetic binding parameters such as on- and off-rates [8]. Thus, we used BLI to determine the binding parameters of TTHA0973 to different DNAs. Experiments with different consensus sequence mutants illustrated the importance of each nucleotide in contributing to TTHA0973-DNA binding specificity. Generally, several single point mutants impacted the dissociation constant only slightly, with the two G/C nucleotides within the palindromic half-site having the most consequential effects. BLI was also performed with the different *T. thermophilus* HB8 genomic sites, to validate whether TTHA0973 binds them avidly. Interestingly, while the binding affinities for these sites generally followed the trends indicated by FIMO, their values varied considerably, with some associations being below detection levels under our experimental conditions. These observations strongly suggest the necessity to perform experimental validation of theoretically determined sites before any conclusions are made. Sakamoto et al. performed a limited biophysical study of TTHA0973-DNA binding, using the related technology, surface plasmon resonance, and dsDNA fragments containing the *TTHA0963* and *TTHA0973* promoter regions [11]. They determined *k*_on_, *k*_off_, and K_D_ values of 9.3 × 10^5^ M^−1^·S^−1^, 1.0 × 10^−3^ s^−1^, and 1.1 nM for *TTHA0963* and 9.8 × 10^5^ M^−1^·S^−1^, 0.9 × 10^−3^ s^−1^, and 0.9 nM for *TTHA0973*, respectively, comparable values to what we obtained. Thus, this provides us with increased confidence in the use of BLI to investigate protein-DNA interactions and the values we obtained for different TTHA0973 binding sites.

For Sakamoto et al., promoter identification and biological role determination for the putative transcription factor TTHA0973 evolved primarily from knowledge about orthologs in other organisms [11]. Thus, they were able to identify TTHA0973 as PaaR, the functional homolog of the transcriptional repressor PaaX found in *E. coli* and *Pseudomonas* strains, which regulates genes involved with phenylacetic acid degradation. While homology comparisons are highly effective approaches for proposing biological functions of proteins, they tend to be directed to a known outcome and are less open to potential discovery. Conversely, our biochemistry-centric approach produced many possible genomic TTHA0973-binding sites, which then had to be winnowed down through bioinformatic analyses and functional studies to yield a set of reasonable candidate genes regulated by TTHA0973. Most important, the two best candidates identified through our studies corresponded to the two gene promoters, *TTHA0963* and *TTHA0973*, identified previously by homology. Our other candidate gene promoter, *TTHA0615*, while acceptable with regards to sequence, intergenic location, proximity to core promoter elements, being first gene in its operon, and experimentally determined binding affinity, is not likely a TTHA0973-regulated gene, as judged from publicly available gene expression profile data comparing wild type and TTHA0973-deficient *T. thermophilus* HB8 strains [13]. Such demonstrates the limitations of a biochemistry-centric approach for identifying putative transcription factor function but also provides guidance as to those parameters that should be gauged most important in making such determinations (*i.e.*, binding affinity) and the need for functional validation whenever possible. With that in mind, one additional gene possessing high affinity TTHA0973 binding sites, *TTHA0647*, was identified in our studies but not pursued based on its location within an open reading frame, distance from potential core promoter elements, and placement within its operon. However, clones containing this sequence were among the most abundant obtained by genomic SELEX, constituting 41 of the 63 clones isolated [11]. In addition, we found two potential TTHA0973 binding sites within 500 bp of one another in this gene, which was unique among the TTHA0973 binding sites we identified. GEO data found no significant change in expression for *TTHA0647* when TTHA0973 was deficient, suggesting it does not play a transcriptional regulatory role for this gene. Thus, while high-affinity TTHA0973 binding sites may be coincidental here, they are worth noting in case they may be involved in an unexpected, DNA-dependent process, as we have observed previously [6].

One concern with a genetic approach for characterizing putative transcription factors is the possibility that the observed changes in gene expression, or lack thereof, may be affected by changes in the activity of endogenous transcription regulators compensating for the loss of the deleted gene product. Thus, upregulation of additional transcription factors involved in regulating phenylacetic acid metabolism could mask identification of the full spectrum of genes controlled by TTHA0973. Given the limited information available on transcription factors in this organism, it is not yet possible to answer whether the expression of any *T. thermophilus* HB8 transcription factor is affected by TTHA0973 depletion. However, it is possible to determine whether the expression of genes encoding the other related TetR-family transcriptional repressors, *TTHA0101*, *TTHA0167*, or *TTHB023*, is affected. Using available GEO data, we found minimal changes in the expression of their transcripts (logFC = 0.025279, 0.195387, and 0.503969, respectively) when TTHA0973 was depleted (see Table S2). These data suggested that none of their genes were significantly upregulated to compensate for the reduction in cellular TTHA0973 levels. Such is notable in that one of these transcription factors, TTHB023, had been previously identified as being involved in phenylacetic acid metabolism and capable of regulating TTHA0973-responsive gene expression in vitro [12]. 

## 4. Materials and Methods

### 4.1. Oligonucleotides

Oligonucleotides used in this study were obtained from Integrated DNA Technologies and are listed in Table S1. The initial REPSA library was prepared by PCR amplification of ST2R24 with primers ST2L and IRD7_ST2R for six cycles, to ensure maximal double-stranded DNA content with fully annealed randomized cassette regions. Subsequent REPSA libraries were PCR amplified for 6, 9, and 12 cycles, to identify the product possessing the aforementioned characteristics. Libraries for massively parallel semiconductor sequencing were prepared by a two-step fusion PCR process, using primers A_BC02_ST2R and trP1_ST2L as the initial set and A_uni and trP1_uni as the second set, as previously described [7]. Other duplex DNAs were prepared by conventional PCR amplification following the manufacturer’s instructions. Those used as EMSA probes were amplified with primers ST2L and IRD7_ST2R, while those used in BLI assays were amplified with primers ST2L and Bio_ST2R.

### 4.2. Protein Expression and Purification

TTHA0973 protein was expressed following IPTG induction of *E. coli* BL21(DE3) bacteria transformed with plasmid pET-ttPaaR and purified from soluble bacterial extracts by heat-treatment as described previously [7]. SDS-PAGE analysis of fractions from a representative purification is shown in Figure S1 and is consistent with a near quantitative recovery of TTHA0973 protein. Analysis by an Agilent P200 ScreenTape assay confirmed that the TTHA0973 preparation used in our studies consisted of a major species of apparent mass 20.6 kDa with greater than 95% purity.

### 4.3. Transcription Factor Consensus Sequence Determination

REPSA selections with 17 nM TTHA0973 were performed essentially as previously described [7], with the exception that the IISRE FokI was used in Rounds 1–3 and BpmI was used in Rounds 4 and 5. Amplicon library preparation, Ion PGM individual sequencing particle (ISP) preparation, Ion PGM semiconductor sequencing, and Ion Torrent sever sequence processing were all performed as previously described [7]. Resulting raw sequences in fastq format (Data S1) were further processed by our Sequencing1.java program [7] and DuplicatesFinder v 1.1 (Available online: http://proline.bic.nus.edu.sg/~asif/tools/DuplicateFinder.zip) to yield data suitable for consensus sequence determination by web version 4.10.2 of Multiple Em for Motif Elicitation (MEME) (Available online: http://meme-suite.org/tools/meme) [18]. Position-weight matrices for the top three motifs were determined and represented as sequence logos, from which a consensus sequence was derived.

### 4.4. Protein-DNA Binding Assays

Electrophoretic mobility shift assays (EMSA) with both libraries and defined DNAs were performed essentially as previously described [7,8], with a detailed protocol being available [19]. Biolayer interferometry was performed essentially as previously described [7], with the exception that only four concentrations of TTHA0973 (11, 33, 100, 300 nM) were used for each DNA probe investigated. Such was sufficient to yield global values for *k*_on_ and *k*_off_ rate constants as well as K_D_ equilibrium binding constants with R^2^ goodness-of-fit determinations of greater than 0.95 in most all cases.

### 4.5. Bioinformatic Determination of Candidate Regulated Genes

An extended 14-bp position weight matrix obtained from a MEME analysis of our processed sequencing data was used as the input to a Find Individual Motif Occurrences (FIMO) analysis (Available online: http://meme-suite.org/tools/fimo) [20], to identify best matches within the *T. thermophilus* genome. Stringency was limited to include matches having *P*-values < 1.25 × 10^–5^. Sequences ±200 bp from the TTHA0973 binding site were analyzed by both Softberry BPROM (Available online: http://www.softberry.com) and University of Groningen Genome2D PePPER (Available online: http://genome2d.molgenrug.nl) to identify potential bacterial core promoter elements [21,22]. Operons were identified using the ProOpDB database at the Universidad Nacional Autónoma de México (Available online: http://biocomputo2.ibt.unam.mx/OperonPredictor/) and data from BioCyc (Available online: http://biocyc.org) [23,24]. Putative biological functions of TTHA0973-regulated genes were obtained using available *T. thermophilus* HB8 databases at KEGG (Available online: https://www.genome.jp/kegg-bin/show_organism?org=T00220) and UniProtKB (Available online: https://www.uniprot.org [300852]) [25,26]. Publicly available microarray data for gene expression profiles in wild-type and TTHA0973-deficient *T. thermophilus HB8* were obtained from the National Center for Biotechnology Information Gene Expression Omnibus (NCBI GEO) website (Available online: https://www.ncbi.nlm.nih.gov/geo/) [15], SuperSeries GSE21875, specifically samples GSM532209, GSM532210, and GSM532211, obtained from wild-type *T. thermophilus* HB8 grown in rich medium for 680 min and samples GSM530126, GSM530127, and GSM530128, obtained from TTHA0973-deficient *T. thermophilus* HB8 strains propagated under identical conditions. These data sets were analyzed using their NCBI GEO2R program with default settings to determine changes in gene expression (LogFC values) and their statistical significance (*P*-values) for our potential TTHA0973-regulated genes.

## Figures and Tables

**Table 1:** TTHA0973-DNA binding parameters.

Name	Sequence	*k*_on_ (M^−1^·s^−1^)	*k*_off_ (s^−1^)	K_D_ (M)	R^2^
wt	AACAAACGTTTGTT	17494	9.446 × 10^−5^	5.400 × 10^−9^	0.9716
m1	tACAAACGTTTGTT	31516	1.422 × 10^−4^	4.512 × 10^−9^	0.9665
m2	AtCAAACGTTTGTT	26873	1.627 × 10^−^^4^	6.054 × 10^−9^	0.9681
m3	AAgAAACGTTTGTT	24948	3.086 × 10^−4^	1.237 × 10^−8^	0.9656
m4	AACcAACGTTTGTT	22865	1.511 × 10^−4^	6.609 × 10^−9^	0.9803
m5	AACAcACGTTTGTT	25404	9.445 × 10^−5^	3.718 × 10^−9^	0.9660
m6	AACAAcCGTTTGTT	27448	1.286 × 10^−4^	4.686 × 10^−9^	0.9440
m7	AACAAAaGTTTGTT	26359	4.372 × 10^−4^	1.659 × 10^−8^	0.9659

(Sequence) Lowercase nucleotides indicate mutation from the consensus TTHA0973 sequence.

**Table 2:** FIMO of the best possible matches to the TTHA0973 consensus sequence.

Start	End	*P*-Value	*Q*-Value	Sequence	Location	Int?	Gene	Operon
229490	229503	3.65 × 10^−6^	1	CACCACCGTTTGTT	+1255	N	*TTHA0236*	5/6
615296	615309	3.65 × 10^−6^	1	ACCTAACGTTCGCT	+733	N	*TTHA0647*	4/4
909145	909158	3.65 × 10^−^^6^	1	AACGGCCGTTAGTT	−46	Y	*TTHA0963*	1/5
917390	917403	3.65 × 10^−6^	1	AACAAACGACCGTT	−3	Y	*TTHA0973*	1/6
220957	220970	5.86 × 10^−6^	1	AAGAAAGGTTAGAT	+175	~	*TTHB214*	2/2
584275	584288	1.02 × 10^−5^	1	AACTAAGGATTGGT	−1	Y	*TTHA0615*	2/3
615792	615805	1.02 × 10^−5^	1	ACCTAGCGTTACTT	+237	N	*TTHA0647*	4/4
59241	59254	1.21 × 10^−5^	1	AACCAGCCTTCCTT	+88	N	*TTHB067*	N
143843	143856	1.21 × 10^−5^	1	AAAGAACCTTCGCT	+131	N	*TTHB153*	N
260398	260411	1.21 × 10^−5^	1	AATGACCCTTGGTT	−41	Y	*TTHA0272*	1/2

(*P*-value) defined as the probability of a random sequence of the same length matching that position of the sequence with an as good or better score. (*Q*-value) False discovery rate if the occurrence is accepted as significant. (Location) location of the TTHA0973-binding site relative to the start site of translation. (Int?) TTHA0973-binding site is located in an intergenic region. (Operon) gene position within the postulated operon. (N) No operon, single transcriptional unit.

**Table 3:** TTHA0973-promoter binding parameters.

Gene	Sequence	*k*_on_ (M^−1^·s^−1^)	*k*_off_ (s^−1^)	K_D_ (M)	R^2^
*TTHA0236*	CACCACCGTTTGTT	100803	8.463 × 10^−4^	8.396 × 10^−9^	0.9631
*TTHA0647*	ACCTAACGTTCGCT	102574	4.044 × 10^−4^	3.943 × 10^−9^	0.9588
*TTHA0963*	AACGGCCGTTAGTT	135763	5.176 × 10^−4^	3.813 × 10^−9^	0.9569
*TTHA0973*	AACAAACGACCGTT	140260	4.286 × 10^−4^	3.056 × 10^−9^	0.9632
*TTHB214*	AAGAAAGGTTAGAT	nab			
*TTHA0615*	AACTAAGGATTGGT	60936	2.300 × 10^−4^	3.774 × 10^−8^	0.9586
*TTHA0647b*	ACCTAGCGTTACTT	38264	3.807 × 10^−4^	9.950 × 10^−8^	0.9741
*TTHB067*	AACCAGCCTTCCTT	Nab			
*TTHB153*	AAAGAACCTTCGCT	95001	2.833 × 10^−4^	2.982 × 10^−7^	0.9018
*TTHA0272*	AATGACCCTTGGTT	nab			

(nab) No apparent binding.

**Table 4:** GEO2R analysis comparing TTHA0973-deficient and wild type *T. thermophilus* HB8 gene expression profiles.

Gene	LogFC	Adj. *P*-Value	*P*-Value	t	B
*TTHA0236*	−0.807	0.132	4.84 × 10^−2^	−2.42	−4.48
*TTHA0647*	−0.386	0.213	1.01 × 10^−1^	−1.90	−5.21
***TTHA0963***	2.09	0.0247	7.84 × 10^−^^4^	5.85	−0.137
***TTHA0973***	6.68	0.00594	1.57 × 10^−5^	11.2	3.80
*TTHB214*	−0.335	0.725	6.05 × 10^−1^	−0.543	−6.68
*TTHA0615*	0.851	0.0539	8.65 × 10^−3^	3.69	−2.68
*TTHB067*	−0.666	0.0695	1.58 × 10^−2^	−3.22	−3.32
*TTHB153*	0.304	0.501	3.55 × 10^−1^	0.994	−6.33
*TTHA0272*	−0.0794	0.870	7.97 × 10^−1^	−0.269	−6.81

(Gene) Bold genes were among the 100 results with the lowest adjusted *P*-values. (LogFC) Log2-fold change between data obtained from TTHA0973-deficient and wild type *T. thermophilus* HB8 strains. (Adjusted *P*-value) *P*-value obtained following multiple testing corrections using the default Benjamini and Hochberg false discovery rate method [14]. (*p*-value) Raw *P*-value. (t) Moderated t-statistic. (B) Log-odds that the gene is differentially expressed.

**Table 5:** TTHA0973-promoter binding parameters.

Promoter	Operon	Gene	Role	LogFC	Adj *P*-Value
~	1	*TTHA0963*	Pseudogene	2.09	7.84 × 10^−4^
	2	*TTHA0965*	phenylacetic acid degradation protein PaaI	2.62	1.00 × 10^−3^
	3	*TTHA0966*	phenylacetyl-CoA ligase	2.41	8.56 × 10^−4^
	4	*TTHA0967*	hypothetical protein	2.58	2.19 × 10^−2^
Y	1	*TTHA0973*	TetR family transcriptional regulator PaaR	6.68	1.57 × 10^−^^5^
	2	*TTHA0972*	phenylacetate-CoA oxygenase subunit PaaA	3.49	1.09 × 10^−5^
	3	*TTHA0971*	phenylacetic acid degradation protein PaaB	3.98	8.23 × 10^−^^7^
	4	*TTHA0970*	phenylacetic acid degradation protein PaaC	4.11	1.81 × 10^−7^
	5	*TTHA0969*	phenylacetic acid degradation protein PaaD	3.74	7.58 × 10^−7^
	6	*TTHA0968*	bifunctional aldehyde dehydrogenase/enoyl-CoA hydratase	4.07	5.78 × 10^−^^7^
Y	1	*TTHA0615*	ATP-dependent Clp protease, protease subunit	0.85	8.65 × 10^−^^3^
	2	*TTHA0616*	ATP-dependent protease ATP-binding subunit ClpX	−0.032	8.74 × 10^−1^

(~) Indicates that while a promoter is present, the TTHA0973-binding site does not overlap core elements (e.g., −35, −10, +1). (2) Number indicates gene position within an operon. (LogFC) Log2-fold change in expression, TTHA0973-deficient:wt *T. thermophilus* HB8 strains, data from GEO [15].

## Supplementary Materials

Supplementary materials can be found at https://www.mdpi.com/1422-0067/20/13/3336/s1.

## Abbreviations

BLI	Biolayer interferometry
EMSA	Electrophoretic mobility shift assay
FIMO	Find individual motif occurrences
GEO	Gene expression omnibus repository
IISRE	Type IIS restriction endonuclease
MEME	Multiple Em for motif elicitation
REPSA	Restriction endonuclease protection, selection, and amplification
SELEX	Systematic evolution of ligands by exponential enrichment
