# Supplementary material for: Identification and Characterization of Preferred DNA-Binding Sites for the Thermus thermophilus HB8 Transcriptional Regulator TTHA0973
Source: Int J Mol Sci. 2019 Jul 7;20(13):3336. doi: 10.3390/ijms20133336 (PMC6651687; doi:10.3390/ijms20133336)
Supplement: Supplementary file 1 [file ijms-20-03336-s001.zip › Table S1.docx]

**Table S1.** Oligonucleotides.

| Name | Sequence | Length | Purif. | Use |
| --- | --- | --- | --- | --- |
| ST2R24 | CTAGGAATTCGTGCAGAGGTGAATNNNNNNNNNNNNNNNNNNNNNNNNTTACCATCCCTCCAGAAGCTTGGAC | 73 | PAGE | REPSA selection template precursor |
| ST2L | CTAGGAATTCGTGCAGAGGTGAAT | 24 | Desalt | PCR primer |
| ST2R | GTCCAAGCTTCTGGAGGGATGGTAA | 25 | Desalt | PCR primer |
| IRD7_ ST2R | /5IRD700/GTCCAAGCTTCTGGAGGGATGGTAA | 25 | HPLC | 5´ IRDye 700-modified PCR primer |
| A_BC02_ST2R | CCATCTCATCCCTGCGTGTCTCCGACTCAGTAAGGAGAACGATGTCCAAGCTTCTGGAGGGATG | 64 | PAGE | Fusion PCR primer |
| trP1_ ST2L | CCTCTCTATGGGCAGTCGGTGATCTAGGAATTCGTGCAGAGGTGA | 45 | PAGE | Fusion PCR primer |
| A_uni | CCATCTCATCCCTGCGTG | 18 | Desalt | PCR primer |
| trP1_uni | CCTCTCTATGGGCAGTCGG | 19 | Desalt | PCR primer |
| ST2_0973_non | AGGAATTCGTGCAGAGGTGAATCAAACGTTCGTTATTACCATCCCTCCAGAAGCTTGG | 58 | Desalt | TTHA0973 nonpalindromic consensus DNA probe precursor |
| ST2_0973_pali | AGGAATTCGTGCAGAGGTGAATTAACGATCGTTATTACCATCCCTCCAGAAGCTTGG | 57 | Desalt | TTHA0973 palindromic consensus DNA probe precursor |
| Bio_ ST2R | /5BiodT/GTCCAAGCTTCTGGAGGGATG | 22 | HPLC | 5´ biotin-modified PCR primer |
| ST2_ REPSAis | CTAGGAATTCGTGCAGAGGTGAATCGTCATAGAATTCGTTACCATCCCTCCAGAAGCTTGGAC | 63 | PAGE | Control DNA precursor |
| ST2_0973_wt | AGGAATTCGTGCAGAGGTGAATAACAAACGTTTGTTTTACCATCCCTCCAGAAGCTTGG | 59 | Desalt | TTHA0973 consensus DNA probe precursor |
| ST2_0973_m1 | AGGAATTCGTGCAGAGGTGAATTACAAACGTTTGTTTTACCATCCCTCCAGAAGCTTGG | 59 | Desalt | TTHA0973 mutant 1 DNA probe precursor |
| ST2_0973_m2 | AGGAATTCGTGCAGAGGTGAATATCAAACGTTTGTTTTACCATCCCTCCAGAAGCTTGG | 59 | Desalt | TTHA0973 mutant 2 DNA probe precursor |
| ST2_0973_m3 | AGGAATTCGTGCAGAGGTGAATAAGAAACGTTTGTTTTACCATCCCTCCAGAAGCTTGG | 59 | Desalt | TTHA0973 mutant 3 DNA probe precursor |
| ST2_0973_m4 | AGGAATTCGTGCAGAGGTGAATAACCAACGTTTGTTTTACCATCCCTCCAGAAGCTTGG | 59 | Desalt | TTHA0973 mutant 4 DNA probe precursor |
| ST2_0973_m5 | AGGAATTCGTGCAGAGGTGAATAACACACGTTTGTTTTACCATCCCTCCAGAAGCTTGG | 59 | Desalt | TTHA0973 mutant 5 DNA probe precursor |
| ST2_0973_m6 | AGGAATTCGTGCAGAGGTGAATAACAACCGTTTGTTTTACCATCCCTCCAGAAGCTTGG | 59 | Desalt | TTHA0973 mutant 6 DNA probe precursor |
| ST2_0973_m7 | AGGAATTCGTGCAGAGGTGAATAACAAAAGTTTGTTTTACCATCCCTCCAGAAGCTTGG | 59 | Desalt | TTHA0973 mutant 7 DNA probe precursor |
| ST2_0973_0236p | AGGAATTCGTGCAGAGGTGAATCACCACCGTTTGTTTTACCATCCCTCCAGAAGCTTGG | 59 | Desalt | *TTHA0236* promoter DNA probe precursor |
| ST2_0973_0647p | AGGAATTCGTGCAGAGGTGAATACCTAACGTTCGCTTTACCATCCCTCCAGAAGCTTGG | 59 | Desalt | *TTHA0647* promoter DNA probe precursor |
| ST2_0973_0963p | AGGAATTCGTGCAGAGGTGAATAACGGCCGTTAGTTTTACCATCCCTCCAGAAGCTTGG | 59 | Desalt | *TTHA0963* promoter DNA probe precursor |
| ST2_0973_0973p | AGGAATTCGTGCAGAGGTGAATAACAAACGACCGTTTTACCATCCCTCCAGAAGCTTGG | 59 | Desalt | *TTHA0973* promoter DNA probe precursor |
| ST2_0973_B214p | AGGAATTCGTGCAGAGGTGAATAAGAAAGGTTAGATTTACCATCCCTCCAGAAGCTTGG | 59 | Desalt | *TTHB214* promoter DNA probe precursor |
| ST2_0973_0615p | AGGAATTCGTGCAGAGGTGAATAACTAAGGATTGGTTTACCATCCCTCCAGAAGCTTGG | 59 | Desalt | *TTHA0615* promoter DNA probe precursor |
| ST2_0973_0647bp | AGGAATTCGTGCAGAGGTGAATACCTAGCGTTACTTTTACCATCCCTCCAGAAGCTTGG | 59 | Desalt | *TTHA0647*b promoter DNA probe precursor |
| ST2_0973_B067p | AGGAATTCGTGCAGAGGTGAATAACCAGCCTTCCTTTTACCATCCCTCCAGAAGCTTGG | 59 | Desalt | *TTHB067* promoter DNA probe precursor |
| ST2_0973_B153p | AGGAATTCGTGCAGAGGTGAATAAAGAACCTTCGCTTTACCATCCCTCCAGAAGCTTGG | 59 | Desalt | *TTHB153* promoter DNA probe precursor |
| ST2_0973_0272p | AGGAATTCGTGCAGAGGTGAATAATGACCCTTGGTTTTACCATCCCTCCAGAAGCTTGG | 59 | Desalt | *TTHA0272* promoter DNA probe precursor |

(N) Random nucleotides. Length is in nucleotides. Promoter DNA probes refer to identified TTHA0973-binding sites therein.
