# Supplementary material for: Identification and Characterization of Preferred DNA-Binding Sites for the Thermus thermophilus HB8 Transcriptional Regulator TTHA0973
Source: Int J Mol Sci. 2019 Jul 7;20(13):3336. doi: 10.3390/ijms20133336 (PMC6651687; doi:10.3390/ijms20133336)
Supplement: Supplementary file 1 [file ijms-20-03336-s001.zip › Figure S1.docx]

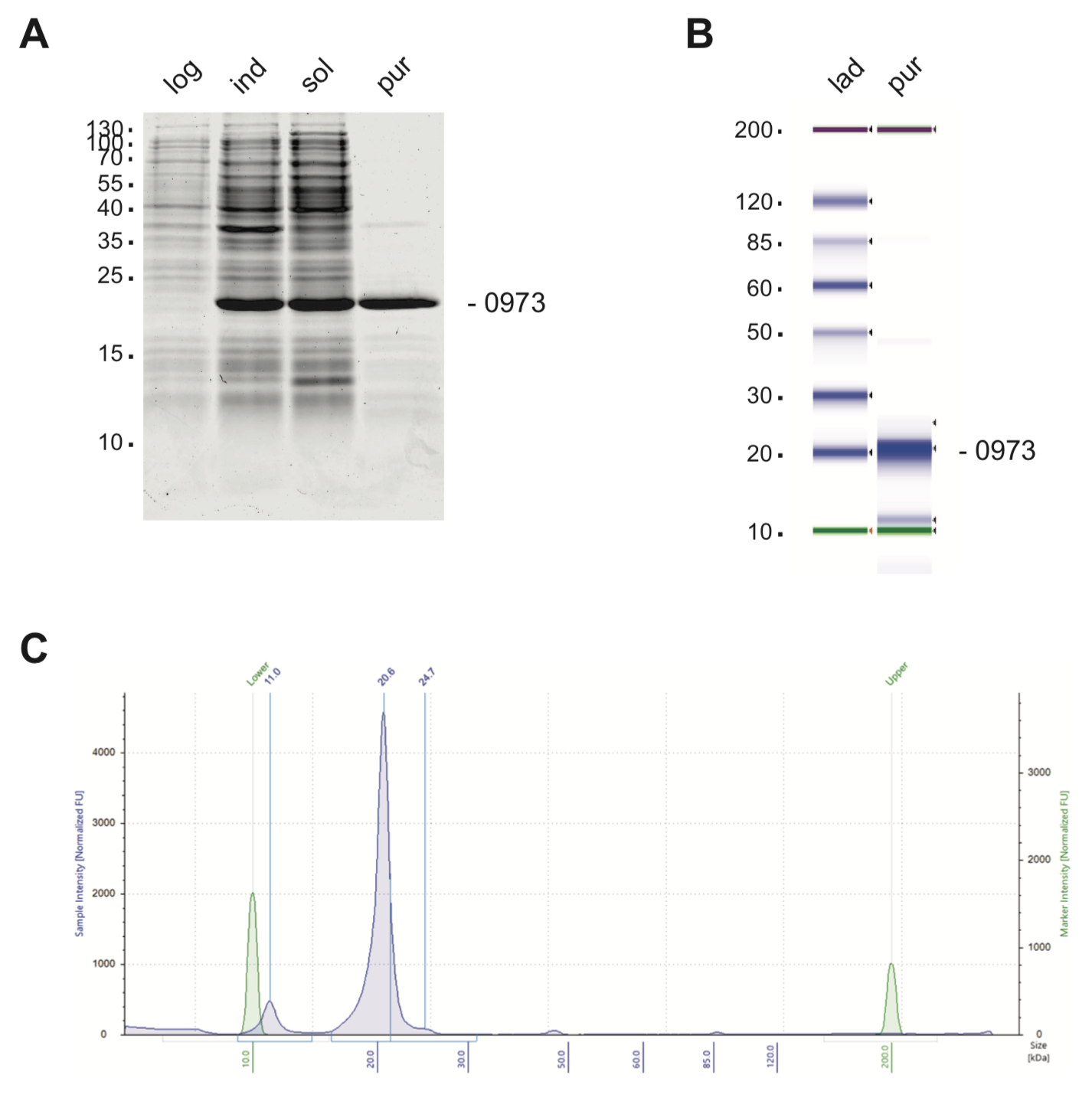


**Figure S1.** Expression and purification of recombinant TTHA0973 protein. (**A**) Shown is an 18% SDS-PAGE gel onto which was loaded whole cell extracts or partially purified fractions equivalent to 0.2% of the total preparation. Lanes shown left to right: (log) logarithmic growth bacteria, (ind) bacteria following IPTG-induction for 4 h, (sol) soluble proteins following sonication and centrifugation, (pur) 4 μg purified TTHA0973 protein. The location of molecular weight standards is indicated at the left of the figure. (**B**) Gel representation of TapeStation P200 data. Lanes shown left to right: (lad) TapeStation 2200 ladder, (pur) 0.2 μg purified TTHA0973 protein. (**C**) Electropherogram of TapeStation P200 TTHA0973 data.
